# The Impact of the Delivery Method on Oxidative Stress in Neonates: A Cross-Sectional Study

**DOI:** 10.3390/jcm14072269

**Published:** 2025-03-26

**Authors:** Barbara Zych, Anna Górka, Aleksander Myszka, Aleksandra Siekierzyńska, Witold Błaż, Dominika Błoniarz

**Affiliations:** 1 Faculty of Health Sciences and Psychology, Medical College, Rzeszow University, Warzywna 1a, 35-310 Rzeszów, Poland; 2 Faculty of Biotechnology, University of Rzeszow, Pigonia 1, 35-310 Rzeszów, Poland; agorka@ur.edu.pl (A.G.); dbloniarz@ur.edu.pl (D.B.); 3 Faculty of Medicine, Medical College, Rzeszow University, Warzywna 1a, 35-310 Rzeszów, Poland; amyszka@ur.edu.pl (A.M.); wblaz@ur.edu.pl (W.B.); 4 Faculty of Medical Sciences and Health Sciences, State Vocational University of Prof. Stanisław Tarnowski in Tarnobrzeg, Sienkiewicza 50, 39-400 Tarnobrzeg, Poland; 5 Department of Physiology and Plant Biotechnology, Faculty of Technology and Natural Sciences, University of Rzeszow, Cwiklińskiej 2, 35-601 Rzeszów, Poland; asiekierzynska@ur.edu.pl

**Keywords:** antioxidant enzymes, Apgar score, bupivacaine, caesarean section, cord blood, oxidative stress, vaginal delivery

## Abstract

**Background/Objectives**: Oxidative stress is a factor that may adversely affect the development of the foetus, the course of labour, and newborn health. This study aimed to determine the association between the labour method, oxidative stress parameters, and neonatal condition. **Methods**: The study material was umbilical cord blood from newborns delivered vaginally (*n* = 60) or by caesarean section (*n* = 108). The total antioxidant status (TAS), superoxide dismutase activity (SOD), glutathione peroxidase activity (GPx), and glutathione reductase activity (GR) were determined using colorimetric methods. The concentration of the chemical elements (Zn, Cu, Mn) was estimated, using atomic absorption spectrometry (ASA). **Results**: The SOD activity was significantly lower in newborns with an Apgar score below 10 at the 5th minute of life compared to newborns with the highest Apgar points (*p* = 0.041). In neonates delivered by caesarean section (CS), but not vaginally born (VB) neonates, the SOD activity was significantly lower in newborns with Apgar scores less than 10 at the 5th minute of life compared to newborns with the maximum number of Apgar points (*p* = 0.02). **Conclusions**: The reduced SOD activity in the umbilical cord blood of newborns with Apgar scores less than 10 could be related to increased oxidative stress during labour. Bupivacaine-induced oxidative stress seems to be the cause of SOD downregulation in caesarean-delivered newborns. The observed SOD downregulation in neonates delivered by CS and with a decreased Apgar score requires confirmation based on a larger cohort of neonates.

## 1. Introduction

Oxidative stress is characterised by increased reactive oxygen species (ROS) activity. It is a consequence of a disturbance in the balance between producing and removing toxic oxygen free radicals, also termed ROS [1]. Thanks to the activity of antioxidants, the human organism tolerates specific amounts of ROS; however, a disturbance in the balance between antioxidant activity and oxidants leads to a violation of the integrity of cells and tissues. This condition can increase apoptosis or necrosis, leading to many diseases [2,3]. Many physiological changes characterise pregnancy. During pregnancy, the mother and foetus consume an increased amount of oxygen and energy substrates. The body’s susceptibility to ROS also increases due to the systemic inflammatory response, which plays a significant role during pregnancy and physiological childbirth [4,5].

Another factor influencing oxidative stress is the pharmacokinetics of medicines, which change during pregnancy due to a reduction in the albumin and Alpha-1-acid glycoprotein (AAG) concentration in plasma and an increase in the free fraction of medicines [6]. This is critical in the case of the administration of bupivacaine, an analgesic most often used in spinal anaesthesia during childbirth [7]. During labour, the causative factor for drug passage across the placenta is the concentration gradient between maternal and foetal blood. The dose, method, and place of administration, and, in the case of a conduit analgesic, additionally, the presence of a vasoconstrictor affects the distribution rate, drug metabolism, and excretion [8,9]. The affinity of bupivacaine to plasma proteins (93–97%), especially to AAG, slightly limits the passage of the drug across the placenta [10]; however, if there is enough time, the drug accumulates in the foetus [9,11], leading to a disturbance of the regulation of redox reactions in cells and cellular dysfunction [12,13], afterwards leading to nervous excitability [14]. The lower AAG concentration in parturients is probably the cause of the reduced binding of bupivacaine. The elevation of AAG in the plasma of pregnant women leads to a concentration of free bupivacaine comparable to that of non-pregnant volunteers. The reduced protein binding of bupivacaine in pregnant women is one of the causes of the higher incidence of toxic reactions to epidural obstetric anaesthesia. The increased toxicity of intravenous infusions of bupivacaine is observed in pregnant animals (ewes) compared to non-pregnant animals. Altered protein binding or hormonal changes are probably responsible for this phenomenon, whereas a local epidural seems to have little influence. However, it could be impactful in inadvertent intravascular injections of local anaesthetics [15].

The progress of labour, supported by uterine contractions and increased intrauterine pressure, leads to periodic and alternating cycles of ischemia and the reperfusion of blood circulating in the uteroplacental unit [16]. During ischemia, increased production of ROS is induced [17,18]. In cells, the primary source of free radicals is mitochondria, where cellular respiration occurs. Oxygen is the final electron acceptor in the mitochondrial respiratory chain, formed by electron transport proteins [19]. Intermittent hypoxemia in eutrophic neonates may be associated with increased oxygen consumption and poor respiratory function (i.e., reduced oxygen uptake by the lung alveoli, reduced oxygen reserves in the lungs, and reduced total oxygen transport capacity of the blood). As a result, hypoxemia may occur in response to very short pauses in breathing, initiating a pathological inflammatory cascade induced by oxidative stress. In animal studies (rodents), hypoxemia has been shown to contribute to generating reactive oxygen species due to NADPH oxidase and induces the accumulation of HIF-1α. Hypoxemia simultaneously reduces HIF-2α levels in carotid bodies and the adrenal medulla, inhibiting superoxide dismutase (SOD2) gene transcription. Thus, hypoxemia initiates the formation of reactive oxygen species, causing a general increase in oxidative stress [20]. Hypoxia-inducible factors (HIF-1α and HIF-2α) control the cellular hypoxic response, activating the expression of selected genes to increase the oxygen supply and reduce oxygen use [21]. Mitochondria are the primary source of ROS in cells, and, at the same time, they are also the primary target of oxidative damage, leading to defects in oxidative phosphorylation and mitochondrial dysfunction. Mitochondrial dysfunction has been implicated in the pathogenesis of bronchopulmonary dysplasia. Moreover, defects in mitochondrial function induced by oxidative stress can exacerbate lung injury [22]. Mitochondrial-derived free radicals have been shown to cause mitochondrial dysfunction in a neonatal hypoxia–ischemia-induced brain injury model. These conditions likely lead to altered gene expression and epigenetic changes that have long-term effects on infant development [22].

Due to its intensive metabolism, the brain, mainly through oxidative metabolism, is particularly susceptible to damage caused by oxidative stress. The brain has low antioxidant activity, removing excessive amounts of free radicals generated during ischemic conditions. The low activity level of catalytic enzymes in the immature brain cannot compensate for excessive superoxide dismutase activity. This condition causes increased hydrogen peroxide production and intensifies brain damage [23]. Moreover, the free oxygen radicals generated during labour and the imbalance between oxidants and antioxidants in the foetus contribute to the occurrence of perinatal and neonatal disorders, such as perinatal asphyxia and hypoxic–ischemic encephalopathy in term infants, bronchopulmonary dysplasia, respiratory distress syndrome, necrotising enterocolitis, especially in premature babies, and sudden infant death syndrome [17,18,24,25]. Compared to full-term newborns, premature infants are more susceptible to ROS damage, because the concentration of antioxidants is low at birth due to a low level of maternal–foetal placental transfer and low endogenous production. Increasing antioxidant synthesis in response to hyperoxia is insufficient in premature infants [26]. Moreover, preterm newborns exhibit a higher level of oxidative stress, which is not only caused by the immaturity of the antioxidant defence system, but is also due to its inability to cope with the oxygen-rich environment and the organs’ immaturity. Medical interventions also contribute to high levels of OS in preterm newborns [27].

Bupivacaine is commonly administered as an anaesthetic during caesarean section. It reduces the permeability of the neuronal cell membrane, blocking the conduction of the action potential [28]. Bupivacaine can cause various adverse effects as a result of an overdose or the accidental intravascular injection of the drug. These changes concern the cardiovascular system, reducing myocardial contractility, causing disorders of automaticity, and a decrease in the conduction velocity of excitations and vasodilation. After the initial phase of stimulation of the central nervous system (CNS) (tachycardia and hypertonia), a phase of depression follows, with the following clinical symptoms: a further decrease in contractility and cardiac output, a drop in blood pressure, severe arrhythmias in the form of ventricular extrasystoles, and even cardiac arrest. The strength of the anaesthetic’s action correlates with the toxic effect on the circulatory system and CNS. However, the cardiotoxic effect of bupivacaine is usually preceded by a reaction from the CNS [28,29]. Neonates exposed, immediately after birth, to higher concentrations of bupivacaine were more likely to be cyanotic and unresponsive to their environment. Their visual abilities and alertness were significantly impaired on the first day after birth and persisted for six weeks [30].

Bupivacaine could disturb mitochondrial oxidative phosphorylation, respiratory chain complexes I and III, and intensify ROS production. ROS over-production activates caspase-3, poly ADP-ribose polymerase degradation, and induces apoptosis in the Schwann cell line [31]. It is predicted that bupivacaine could induce mitochondrial oxidative stress through phosphating CaMK2α, which activates CREB–MCU (cAMP response element-binding protein–mitochondrial Ca^2+^ uniporter) signalling. The CaMK2α–MCU–mitochondrial oxidative stress pathway is a primary mechanism whereby bupivacaine induces neurotoxicity [31].

According to the literature, anaesthesia with the addition of fentanyl reduces the required dose of the anaesthetic by up to 25%, and lowering the dose of the anaesthetic reduces the risk of toxicity and the frequency and severity of the motor blockade. The combination of bupivacaine with an opioid reduces the MED (median effective dose); moreover, increasing the concentration of the opioid (fentanyl) decreases the MLAC (minimum local analgesic concentration) [32].

Most studies focus on the effect of anaesthesia on the mother’s and newborn’s disease and premature babies’ growth and development. It is well-documented that obstetric general anaesthesia agents are associated with increased risks of tachycardia, hypertension, and respiratory adverse events in infants, including coughs and laryngospasms [33]. General anaesthesia also tends to depress Apgar scores at 1 min [34]. However, there is a lack of research on the impact of anaesthesia on oxidative stress in low-risk (full-term) pregnancies.

Given the lack of research on oxidative stress in full-term neonates exposed to anaesthesia, this study aims to evaluate oxidative stress markers in newborns delivered via vaginal birth (without anaesthesia) versus caesarean section (with spinal anaesthesia). Understanding these effects is critical, due to the increasing prevalence of C-sections and the unavoidable use of anaesthesia in these cases.

## 2. Materials and Methods

### 2.1. Study Design

Our study utilises a cross-sectional study design, analysing umbilical cord blood samples to identify the antioxidant status and trace element concentrations. We present the results from examining the association between delivery methods, the Apgar score, and oxidative stress parameters in neonates.

This study used the Apgar scale, based on five features, namely pulse, reflex irritability, muscle tone, respiration, and skin colour at one, three, five, and ten minutes after birth [35]. Anaesthesiologists traditionally use these five parameters to monitor the patient’s condition. The Apgar scale is used as a rapid indicator in the assessment of the newborn’s condition immediately after birth and in response to resuscitation [36]. The number of points obtained by a newborn depends in part on the physiological maturity of the newborn [37]. The Apgar score shows differences between newborns whose mother received local (spinal subarachnoid) or general anaesthesia for a caesarean section, but it is not specifically designed to assess the effects of anaesthesia on the newborn [37].

The criteria for inclusion in the study were newborns associated with full-term (low-risk) pregnancy (between 38–41 weeks of gestation). In the group of pregnancies ending in vaginal delivery, the progress of labour was determined based on an internal (vaginal) examination every two hours or in accordance with clinical indications. Uterine contractions and the foetal heart rate were continuously monitored using a cardiotocograph until delivery, in all pregnancies.

The exclusion criteria for the study were mothers with diseases, including diabetes, hypertension, heart disease, and kidney disease, as well as mothers with multiple pregnancies, foetal developmental defects, anaemia in the mother or foetus, and smoking.

### 2.2. Setting and Participants

The study group comprised 168 pregnant women, who gave birth in the Department of Gynecology and Obstetrics in Clinical Hospital No. 2, Saint Jadwiga Queen in Rzeszów, Poland.

### 2.3. Biological Material Collection

Venous umbilical cord blood samples were collected from the umbilical vessel immediately after delivery, using vacuum syringe tubes containing EDTA and lithium heparin (Sarstedt, Nümbrecht, Germany). After collection, the sample was transported to the laboratory in a transport refrigerator at +4 °C within 15 min. In the laboratory, the samples were stored at +4 °C. Up to a maximum of 60 min after delivery, the samples were centrifuged at 2500 rpm for 5 min at +4°C (SIGMA 2-16PK; Sigma Laborzentrifugen GmbH, Osterode am Harz, Germany) to separate the plasma from the blood cells. The plasma samples were stored at −80 °C until the biochemical analyses. After thawing, 1000× *g* of the sample was centrifuged for 1 min.

### 2.4. Analysis of Oxidative Stress Parameters

The total antioxidant status (TAS), glutathione peroxidase (GPx) activity, glutathione reductase (GR) activity, and superoxide dismutase (SOD) activity in blood serum were assessed using colorimetric methods, using kits from Randox (Randox Laboratories Ltd., Crumlin, UK), according to the manufacturer’s instructions. Dedicated controls were used to monitor the assays using biochemistry analysers. The reaction mixture was prepared in 24-well plates (JET BIOFIL; Guangzhou Jet Bio-Filtration Co., Ltd., Guangzhou, China). Absorbance measurements were performed using a Tecan Infinite M200 Pro spectrophotometer and Magellan 7.1 software (Tecan Group Ltd., Männedorf, Switzerland).

The concentration of SOD cofactors, zinc, copper, and manganese, was determined using atomic absorption spectrometry (ASA), as described previously [38].

### 2.5. Statistical Analysis

Statistical analysis was conducted using the Statistica software v.13 (StatSoft, Kraków, Poland). Nonparametric tests were used to assess statistically significant differences between the groups. Meeting certain assumptions was assessed using the following statistical tests: the homogeneity of variance was analysed using Levene’s test, and testing for normality was performed using the Shapiro–Wilk test. Pearson’s or Spearman’s correlation was adopted to evaluate the correlations between the analysed variables. Moreover, *p*-values < 0.05 were considered statistically significant. Principal Component Analysis (PCA) was used to observe the trends in regard to the oxidative stress parameters. We also wanted to uncover the correlations between the variables. The number of components retained was determined using a scree plot. PCA was performed using the R Package v.4.3.1. (R Foundation for Statistical Computing, Vienna, Austria).

## 3. Results

### 3.1. Study Participants

The obstetrics and gynaecology history and medical records of the participants were obtained from a questionnaire completed by the mothers and during an interview. The average age of the studied women was 30 years. For most of them, it was their second labour (I: *n* = 56, 33.30%; II: *n* = 65, 38.70%; III: *n* = 47, 28.00%), ending with a caesarean section (vaginal birth (VB): *n* = 60, 35.70% vs. caesarean section (CS): *n* = 108, 64.30%), predominantly during the 39th week of pregnancy (39.29 ± SD 0.95). In the group of parturient women whose delivery ended with a caesarean section, intrapartum indications predominated (50%), including a lack of labour progress (37%), foetal heart rate disturbances (13%), and a non-cephalic foetal position (5%). Other causes of the surgical delivery were non-obstetric indications, delivery after a previous caesarean section in 17.0% of cases, as well as the patient’s lack of consent for a vaginal delivery (11%). All the women that gave birth by caesarean section received spinal anaesthesia. After the initial hydration of the parturients’ body with intravenous Ringer’s solution, 2.0 mL of 0.5% bupivacaine, with the addition of approximately 10 μg of fentanyl, was administered intrathecally. The morphological parameters of the blood of the pregnant women were within the reference values. The average duration for particular stages in vaginal deliveries is presented in Table 1.

In the study group, newborns from uncomplicated pregnancies obtained a high number of points (1st minute: 9.80 ± 0.81 points, 3rd minute: 9.91 ± 0.49 points, 5th minute: 9.92 ± 0.38 points, 10th minute: 9.97 ± 0.24 points) regardless of the method of the termination of the pregnancy, the anthropometric factors, and the clinical parameters (Table 2 and Table 3).

### 3.2. Oxidative Stress in Newborns Considering the Delivery Method

The activity of TAS, SOD, GPx, and GR did not differ significantly between newborns delivered vaginally and those delivered by caesarean section. The concentration of elements that are SOD cofactors did not differ significantly between the newborns regardless of the delivery method (Table 4).

The results showed a negative correlation (Pearson’s r = −0.17, *p* = 0.021) between the SOD activity and Cu concentration in umbilical cord blood. This correlation was also observed in VB newborns (Pearson’s r = −0.33, *p* = 0.007).

### 3.3. Oxidative Stress in Newborns Considering the Apgar Score

The analysis of the oxidative stress parameters and the Apgar score assessed at the 1st and 3rd minute of the newborns’ life showed no significant differences in the oxidative stress parameters between the newborns with an Apgar score of 10 and those with a score below 10 (Table 5). However, the SOD activity differed significantly between the newborns with an Apgar score of 10 and those with a score below 10 at the 5th minute of the newborns’ life (*p* = 0.041) (Table 5).

The oxidative stress parameters are presented for the newborns with an Apgar score assessed at the 1st, 3rd, and 5th minute of life (Table 6 and Table 7). The oxidative stress parameters in neonates with an Apgar score that was evaluated in the 10th minute of life were not analysed, due to presence only single cases in a subgroup with an Apgar score below ten.

### 3.4. Oxidative Stress in the Subgroup of Newborns Delivered Vaginally Considering the Apgar Score

The oxidative stress parameters in newborns delivered vaginally presented no statistically significant differences between newborns with an Apgar score less than 10 and those with 10 pts at the 1st, 3rd, and 5th minute of life (Table 6).

### 3.5. Oxidative Stress in the Subgroup of Newborns Delivered by Caesarean Section Considering the Apgar Score

The analysis of the oxidative stress parameters in newborns delivered by caesarean section showed no statistically significant differences between the 1st and 3rd minute of life. In the group of newborns delivered by caesarean section, the SOD activity differed significantly between newborns with an Apgar score of 10 and newborns with a lower Apgar score at the 5th minute of life (Table 7).

### 3.6. Principal Component Analysis (PCA) of Parameters Influencing Neonatal Conditions

**Figure 1:**
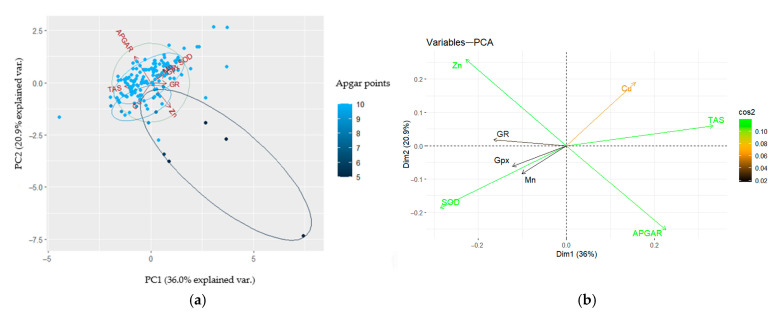
Principal Component Analysis based on oxidative stress markers in newborns and the Apgar score at the 1st minute of life; (**a**) score plot of cases and grouping of variable ellipses. Each dot represents a case, and the shade of the colour reflects the variable’s value. (**b**) Variable correlation plots. Variables positively correlated are collected together, and those negatively correlated are located on opposite sides of the plot. The distance between the variables and the origin reflects the quality of the variables (marked as cos2), and the colour reflects the value of the variables.

The cord blood antioxidant parameters were analysed using Principal Component Analysis (PCA) to identify the oxidative stress markers and their cofactors, influencing the clinical condition of newborns (Figure 1a,b, Table 8). The most essential elements in regard to the first component were TAS and SOD. The TAS and SOD parameters were negatively correlated. The main elements of the second component were the Apgar score, Zn, Cu, and Mn. The analysed variables negatively correlated with the Apgar score, whereas Mn positively correlated with the Apgar score. The first component accounted for 36.0% of the variation; the second component accounted for 20.9% of the variation (Figure 1a). This led to the identification of clusters of oxidative stress parameters that had a strong (green), medium (orange), and low (brown) impact on the newborn’s Apgar score (Figure 1b). The first principal component correlates most strongly with the TAS and SOD parameters. The second principal component correlates with Cu, Zn, and the Apgar score (Table 8).

## 4. Discussion

There are many causes of the induction of oxidative stress during physiological labour. Oxygen consumption increases during pregnancy and labour, resulting in increased mitochondrial respiration and the release of free electrons in the electron transport chain, stimulating the formation of reactive oxygen species (ROS). In addition, during physiological labour, uterine contractions cause an increase in intrauterine pressure periodically. This results in the obstruction of uteroplacental blood flow and alternating cycles of ischemia and reperfusion. The rapid change from relative intrauterine hypoxia to an extrauterine environment, where O2 pressure is higher, also contributes to oxidative stress [16]. Increased levels of inflammatory cytokines and chemokines and the activation of leukocytes in the uterine myometrium, decidua, cervix, and peripheral blood characterise labour. This results in increased uterine contractility, the activation of decidua/foetal membranes, and maturation of the cervix. Foetal paracrine inflammatory signalling via extracellular vesicles may induce changes in maternal cells to coordinate the ROS-mediated labour process. Exosomes and MVs (microvesicles), released from maturing foetal tissues at term, induce paracrine signalling and increase the production of inflammatory cytokines (IL-6, IL-8, and GM-CSF), which contribute to inflammatory changes in maternal tissues and induce labour [39]. The mode of delivery impacts the release of cortisol (a hyperglycaemic hormone) and oxidative stress in newborns. Higher cortisol levels characterise newborns born by VB compared to newborns delivered by CS. This condition in newborns born vaginally may be related to the more stressful mode of delivery for the child [40].

In our study, we analysed the associations between oxidative stress parameters (TAS, SOD and Cu/ZnSOD and MnSOD, GPx, GR) in newborns born from full-term uncomplicated pregnancies and the delivery method (vaginal birth without anaesthesia vs. caesarean section under anaesthesia with subarachnoid bupivacaine). We also assessed the associations between the mentioned oxidative stress parameters and the Apgar score, based on a clinical evaluation in the first minutes of the newborn’s life. The SOD activity was significantly lower in the group of newborns with an Apgar score below 10, compared to newborns with the highest Apgar score at the 5th minute of life (*p* = 0.041). The lower SOD activity observed in the umbilical cord blood of newborns with Apgar scores less than ten may be an indicator of excessive ROS production, intensifying oxidative stress. However, it may also result from the inactivation of enzymes involved in free radical processes, due to the depletion of enzyme activity [41,42] or changes in the concentration of their cofactors [43]. Copper is one of the cofactors of copper–zinc superoxide dismutase, which eliminates ROS. Low copper content may affect the decrease in enzyme activity and change the rate of synthesis of catalase and Mn-SOD, potentially affecting neurodegenerative disorders in newborns [44].

Concerning the delivery method, we showed that in the subgroup of newborns delivered by CS, the SOD activity was significantly lower in newborns with an Apgar score below 10, compared to newborns with the maximum Apgar score, in the 5th min of life (*p* = 0.02). Our findings are consistent with the findings by Sajjad et al. [42], who also observed altered SOD levels in CS-delivered neonates. However, while their study reported higher SOD levels in CS neonates, we found decreased SOD activity in those with lower Apgar scores, indicating a potential role of oxidative stress in neonatal adaptation.

There is growing evidence that bupivacaine induces oxidative stress. In vitro and in vivo studies have indicated that the over-production of reactive oxygen species is caused by bupivacaine [3,45]. Moreover, it has been shown that bupivacaine treatment led to a significant decline in SOD activity in human neuroblastoma cells [46] and mouse dorsal root ganglia neurons [47] and myoblast cells [48]. It is also well-known that during caesarean section, bupivacaine penetrates newborn blood. The umbilical vein to the maternal vein concentration ratio of unbound bupivacaine was assessed as 0.69 [49]. This phenomenon could be the cause of the decreased SOD activity in umbilical cord blood observed in our study. Considering the observed SOD downregulation in caesarean-delivered newborns and the reports in the literature that bupivacaine treatment led to a substantial decline in SOD activity [46,47,48], we conclude that bupivacaine could induce oxidative stress in neonates during caesarean section deliveries. Our assumption supports the studies published by Rosenblatt and their team, wherein bupivacaine was administered. Immediately after caesarean section, they observed cyanosis more often in in newborns, and their visual perception and alertness were significantly impaired [30].

In our study, PCA analysis showed that SOD activity was associated with the manganese concentration. Manganese is a component of enzymes from the superoxide dismutase group, which eliminates free oxygen radicals [50]. Manganese activates glycosyltransferase and other manganese-dependent enzymes, such as arginase, phosphatase, cholinesterase, and pyruvate carboxylase. One of the most important functions of manganese is its connection with antioxidant processes. As a component of superoxide dismutase (Mn-SOD), it eliminates free oxygen radicals formed during metabolic transformations of the cell. Both a deficiency and excess manganese cause disorders in the body’s functioning [51]. So far, few studies have described the role of this element in umbilical cord blood and its impact on foetal development [52,53]. Zhou et al. [53] revealed that in low-risk pregnancies (37–41 weeks of pregnancy), lower concentrations of manganese in the umbilical cord blood of newborns concerning the mother’s venous blood shows a probable correlation with the increase in oestrogen levels during pregnancy, which stimulates the growth of smooth muscles in the uterus, preparing them for labour contractions, and by affecting the connective tissue in the pelvic area, it facilitates the childbirth process [54]. In turn, Lazer et al. [55] showed significantly higher levels of Mn ions in the umbilical artery of newborns born vaginally than by caesarean section. This phenomenon is presumably related to the regulatory role of MnSOD in initiating full-term labour and oxidative stress [56]. It turns out that the method of ending labour also causes a specific distribution of zinc between the blood plasma and internal organs, moving it from the circulation to the liver and other tissues [57], but it may also be a non-specific reaction of the body to stress caused by an increase of cortisol in the blood [58]. However, we observed a slightly higher zinc concentration in the umbilical cord blood of vaginally delivered newborns in the group with a lower Apgar score (<10 points). Our previous studies showed a 11.2% lower zinc concentration in the mother’s venous blood plasma compared to umbilical cord blood [38]. A copper deficiency reduces the activity of some enzymes, i.e., zinc–copper superoxide dismutase Cu/ZnSOD, ceruloplasmin, catalase and glutathione peroxidase, and affects the proper functioning of free radical scavengers, i.e., metallothionein and glutathione [59]. Our observations showed no association between the pregnancy termination method, the Apgar score, and the copper concentration. However, Grzeszczak et al. showed a positive correlation between the Cu concentration and SOD activity (r = 0.50, *p* <0.001) in umbilical cord blood [60]. The studies mentioned above indicate that the concentrations of the tested trace elements could play an essential role during delivery.

Glutathione peroxidase (GPx) is another antioxidant enzyme that primarily reduces hydrogen peroxide and organic peroxides with the participation of reduced glutathione (GR) [61], which can be excreted from erythrocytes during long-term oxidative stress [62]. In our studies, we observed a slightly higher GPx activity in neonates born vaginally and with a maximum Apgar score compared to children born by caesarean section and with a lower Apgar score. Our results are consistent with other authors’ observations [62,63]. Georgeson et al. [63] showed a higher GPx activity in newborns from low-risk pregnancies that ended with a vaginal delivery compared to caesarean section. Raijmakers et al. [62] also observed higher levels of glutathione in arterial umbilical cord blood after a vaginal birth than after a caesarean section (VB: 3.5 [0.6–22.7] vs. CS: 2.3 [0.7–24.3] μmol/L, *p* < 0.02), indicating that vaginal delivery is associated with more oxidative stress than during delivery by caesarean section. During labour, periods of hypoxia and oxidative stress may explain the higher oxidative stress in vaginally delivered neonates, as the plasma contains a low concentration of antioxidant components to counteract oxidative damage [64]. Lurie et al. [65] showed that increased GPx activity occurred in newborns delivered by emergency caesarean section compared to newborns delivered by elective caesarean section [65]. It also turns out that these newborns had lower GPx values in the umbilical vein and artery concerning the mother’s venous blood before and after birth (*p* < 0.05) [16].

The literature reports a high susceptibility of foetuses to oxygen damage due to the incomplete maturity of antioxidant systems and the high energy demand of tissues during childbirth [66]. Therefore, in our study, we also analysed the total antioxidant capacity in umbilical cord blood of newborns and observed slightly higher TAS levels in the group of VB neonates with a lower Apgar score (<10 points) than in newborns delivered by CS section and with 10 Apgar points. The reduction in TAS in newborns delivered by CS with a maximum Apgar score may be the result of bupivacaine penetrating the foetus through the placenta (30–40% of the concentration in the mother’s body) [10]. Díaz-Castro’s team [16] found similar differences between the umbilical vein and the artery in low-risk pregnancies. Yalcin et al. [67] described a significantly increased total antioxidant capacity TAC in the umbilical artery when mothers receive preoperative oxygen (*p* = 0.003). They also showed that an increased total oxidative status (TOS) and oxidant status index (OSI) occurred in regard to the umbilical arteries of newborns of mothers breathing atmospheric air before delivery (CS with spinal anaesthesia) (*p* = 0.02 and *p* <0.001, respectively). However, Parmigiani et al. [68] showed no differences between vaginal deliveries and caesarean section. Other research [69] has indicated that TAS and TOS may increase during oxytocin-induced deliveries compared to spontaneous deliveries.

The main limitation of our study is the low number of parturient women recruited to participate in the study. The small number of cases in some subgroups (e.g., newborns VB and neonates with a low Apgar score) limited the detection of associations. More studies on larger groups of neonates are required to confirm the associations revealed. Another limitation of our study is that not all of the possible oxidative stress markers were analysed. Analysis of a broader range of markers could enable the identification other markers associated with oxidative stress during labour.

## 5. Conclusions

Considering the delivery method, this study provides new insight into oxidative stress parameters in regard to umbilical cord blood in low-risk pregnancies. This report provides valuable insights into oxidative stress in neonates during labour in physiological and anaesthesia conditions. Selected oxidative stress markers were studied during delivery, including the implications for neonates’ clinical condition based on the Apgar score. We found an association between the activity of SOD in umbilical cord blood and the Apgar score. The decrease in the activity of SOD during labour could result from excessive ROS production and the severity of oxidative stress. However, it may also result from the inactivation of the enzymes involved in antioxidant processes or changes in the status of trace elements that are their cofactors. This may be especially the case since low levels of trace elements, such as Mn, may be a factor in reducing the catalytic activity of SOD and, thus, intensifying the level of oxidative stress, which adversely affects the clinical condition of newborns. Reduced SOD activity in the umbilical cord blood of newborns could be related to bupivacaine-induced oxidative stress. Enhancing the antioxidant capacity in pregnant women might play a substantial role in reducing the bupivacaine-induced oxidative stress consequences during labour. The observed SOD downregulation in neonates delivered by CS and with a decreased Apgar score requires confirmation using a larger cohort of neonates to support the reliability of this phenomenon.

Oxidative stress contributes to many neonatal disease states; therefore, explaining the pathomechanism of oxidative damage in newborns associated with the labour method is crucial. Our study identified SOD as a biomarker associated with a lower Apgar score in CS neonates. This finding underscores the significance of this biomarker in neonate complication risk assessments and in regard to delivery-related conditions. This review fills the knowledge gap related to oxidative stress during physiological delivery and under anaesthesia. The results are a valuable resource for researchers and healthcare professionals interested in newborn healthcare, paving the way for further investigations into the implications of oxidative stress complications induced by medical interventions. Our findings highlight the significance of excess oxidative stress in neonates under anaesthesia conditions and the need to avoid factors that increase oxidative stress if it is not absolutely necessary.

It is justified to continue research that clarifies the issue of the impact of oxidative stress parameters on newborn health, especially in regard to such aspects as long-term follow-up studies to assess the effects of oxidative stress on neonatal development, comparative studies with different anaesthetics to evaluate their effects on oxidative stress, or investigations into maternal antioxidant levels and their influence on neonatal outcomes. Further research on oxidative stress in newborns may also contribute to developing laboratory tests to assess the risk of birth complications and health conditions or disorders in newborns.

## Figures and Tables

**Table 1:** Characteristics of pregnancy and delivery duration.

Parameter	$\bar{x}$ ± SD	Median	Min–Max
Women’s age (years)	30.32 ± 5.44	30	26–34
Completed week of gestation	39.29 ± 0.95	39	39–40
Duration of vaginal birth (minutes)			
I stage of labour	343.79 ± 220.5	285	4–1045
II stage of labour	20.08 ± 23.35	5	1–85
III stage of labour	3.93 ± 2.23	5	1–15

**Table 2:** Anthropometric characteristics and clinical parameters of all the newborns.

Parameter	$\bar{x}$ ± SD	Median	Min–Max
Birth weight (g)	3454.46 ± 463.21	3490.00	2160.00–4830.00
Body length (cm)	55.39 ± 2.60	56	48–63
Head circumference (cm)	34.26 ± 1.66	34	26.00–39.00
Apgar score (pts)			
At 1st minute	9.80 ± 0.81	10	5–10
At 3rd minute	9.91 ± 0.49	10	6–10
At 5th minute	9.92 ± 0.38	10	7–10
At 10th minute	9.97 ± 0.24	10	7–10

**Table 3:** Comparison of newborn parameters delivered by vaginal birth and by caesarean section.

	Vaginal Birth (*n* = 60)			Caesarean Section (*n* = 108)			
Parameter	$\bar{x}$ ± SD	Median	Min–Max	$\bar{x}$ ± SD	Median	Min–Max	*p*-Value
Birth weight (g)	3397.36 ± 432.52	3365.00	2420.00–4340.00	3488.25 ± 480.15	3525.00	2160.00–4830.00	0.161
Body length (cm)	55.41 ± 2.49	55	48–63	55.41 ± 2.67	56	48–62	0.810
Head circumference (cm)	33.82 ± 1.29	34	31.00–36.00	34.51 ± 1.82	35	26.00–39.00	0.004
Apgar score (points)							
At 1st minute	9.86 ± 0.65	10	5–10	9.81 ± 0.79	10	5–10	0.834
At 3rd minute	9.89 ± 0.46	10	7–10	9.91 ± 0.49	10	6–10	0.849
At 5th minute	9.89 ± 0.46	10	7–10	9.95 ± 0.32	10	7–10	0.689
At 10th minute	9.96 ± 0.35	10	7–10	9.99 ± 0.09	10	9–10	0.952

**Table 4:** Oxidative stress parameters in newborns delivered vaginally and by caesarean section.

	Vaginal Birth (*n* = 60)			Caesarean Section (*n* = 108)			
Parameter	$\bar{x}$ ± SD	Median	Min–Max	$\bar{x}$ ± SD	Median	Min–Max	*p*-Value
TAS [mmol/L]	1.81 ± 0.30	1.84	1.24–2.38	1.76 ± 0.34	1.72	1.01–2.83	0.246
SOD [U/mL]	192.57 ± 18.06	184.71	160.53–224.75	196.98 ± 17.89	200.72	162.78–281.41	0.222
Mn [µmol/L]	0.09 ± 0.10	0.07	0.003–0.62	0.14 ± 0.21	0.08	0.01–1.81	0.084
Cu [µmol/L]	6.91 ± 3.39	6.90	0.10–15.50	7.03 ± 4.67	6.30	0.90–33.40	0.453
Zn [µmol/L]	15.62 ± 8.72	13.20	3.20–42.40	13.58 ± 8.60	11.80	1.20–50.40	0.064
GPx [U/g Hg]	154.08 ± 108.64	144.68	93.41–1005.02	149.75 ± 91.35	140.46	96.92–1057.14	0.810
GR [U/L]	86.48 ± 39.61	83.58	1.60–185.29	82.09 ± 38.69	73.26	1.24–191.69	0.484

**Table 5:** Oxidative stress parameters in regard to the newborns’ umbilical cord blood and the newborns’ clinical condition (Apgar score) at the 1st, 3rd, and 5th minute of life.

	Apgar Score in 1st Minute of Life of Newborns (*n* =168)						
	<10 pts (*n* = 17)			10 pts (*n* =151)			*p*-Value
Parameter	$\bar{x}$ ± SD	Median	Min–Max	$\bar{x}$ ± SD	Median	Min–Max	
TAS [mmol/L]	1.84 ± 0.42	1.77	1.18–2.63	1.78 ±0.32	1.76	1.01–2.83	0.667
SOD [U/mL]	189.75 ± 17.00	180.47	167.00–217.60	195.49 ± 18.63	197.91	132.24–281.41	0.358
Mn [µmol/L]	0.09 ±0.08	0.07	0.02–0.36	0.12 ± 0.19	0.08	0.003–1.81	0.726
Cu [µmol/L]	7.28 ± 1.82	7.80	4.30–10.40	6.92 ± 4.41	6.40	0.10–33.40	0.226
Zn [µmol/L]	23.83 ± 33.03	14.50	2.40–143.90	14.15 ± 8.68	12.30	1.20–50.40	0.242
GPx [U/g Hg]	136.72 ± 18.94	130.63	96.92–168.56	152.78 ± 101.21	143.27	93.41–1057.14	0.258
GR [U/L]	89.01 ± 29.06	79.04	31.65–145.81	82.86 ± 39.54	74.33	1.24–191.69	0.303
	**Apgar Score in 3rd Minute of Life of Newborns (*n* = 168)**						
	**<10 pts (*n* = 11)**			**10 pts (*n* = 157)**			***p*** **-Value**
**Parameter**	$\bar{x}$ **± SD**	**Median**	**Min–Max**	$\bar{x}$ **± SD**	**Median**	**Min–Max**	
TAS [mmol/L]	1.87 ±0.34	1.79	1.46–2.31	1.78 ± 0.32	1.76	1.01–2.83	0.459
SOD [U/mL]	189.13 ± 18.91	179.22	167.00–217.60	195.07 ± 18.50	196.36	132.24–281.41	0.384
Mn [µmol/L]	0.09 ± 0.10	0.05	0.02–0.36	0.12 ± 0.18	0.08	0.003–1.81	0.263
Cu [µmol/L]	6.87 ± 1.97	6.40	4.30–10.40	6.95 ± 4.32	6.50	0.10–33.40	0.728
Zn [µmol/L]	31.64 ± 43.62	16.90	2.40–143.90	14.19 ± 8.53	12.30	1.20–50.40	0.518
GPx [U/g Hg]	131.60 ± 19.71	129.58	96.92–155.92	152.06 ± 99.13	143.27	93.41–1057.14	0.150
GR [U/L]	87.29 ± 29.26	88.20	31.65–133.19	83.22 ± 39.28	74.68	1.24–191.69	0.529
	**Apgar Score in 5th Minute of Life of Newborns (*n* = 168)**						
	**<10 pts (*n* = 8)**			**10 pts (*n* = 160)**			***p*** **-Value**
**Parameter**	$\bar{x}$ **± SD**	**Median**	**Min–Max**	$\bar{x}$ **± SD**	**Median**	**Min–Max**	
TAS [mmol/L]	1.85 ±0.37	1.87	1.32–2.31	1.78 ±0.32	1.76	1.01–2.83	0.569
SOD [U/mL]	182.18 ± 12.74	178.53	167.00–210.56	195.61 ±18.58	198.38	132.24–281.41	0.041
Mn [µmol/L]	0.07 ± 0.04	0.07	0.02–0.13	0.12 ± 0.18	0.07	0.003–1.81	0.406
Cu [µmol/L]	7.28 ± 1.53	7.40	4.90–9.50	6.93 ± 4.34	6.40	0.10–33.40	0.327
Zn [µmol/L]	15.04 ± 6.27	13.00	7.60–28.30	14.96 ± 13.17	12.30	1.20–143.90	0.503
GPx [U/g Hg]	140.64 ±19.27	139.06	111.67–168.56	151.63 ±99.21	142.92	93.41–1057.14	0.772
GR [U/L]	81.13 ±26.30	75.93	31.65–124.47	83.55 ±39.40	74.68	1.24–191.69	0.865

**Table 6:** Oxidative stress parameters in regard to the umbilical cord blood of newborns delivered by vaginal birth (VB) and the clinical condition of the newborn (Apgar score at 1st, 3rd, and 5th minute of life did not change).

	Apgar Score of Newborns Delivered by VB (*n* = 60)						
	<10 pts (*n* = 5)			10 pts (*n* = 55)			*p*-Value
Parameter	$\bar{x}$ ± SD	Median	Min–Max	$\bar{x}$ ± SD	Median	Min–Max	
TAS [mmol/L]	1.87± 0.27	1.87	1.51–2.22	1.81 ±0.30	1.84	1.24–2.38	0.624
SOD [U/mL]	186.40 ± 14.11	179.23	176.59–210.56	192.98 ± 18.22	185.00	160.53–224.75	0.542
Mn [µmol/L]	0.09 ±0.04	0.10	0.02–0.13	0.09 ± 0.10	0.07	0.003–0.624	0.603
Cu [µmol/L]	7.36 ± 1.32	8.00	5.20–8.70	6.88 ± 3.49	6.40	0.10–15.50	0.704
Zn [µmol/L]	15.40 ± 3.46	14.75	11.80–20.30	15.64 ± 8.96	13.20	3.20–42.40	0.596
GPx [U/g Hg]	141.52 ± 18.33	136.60	124.31–168.56	154.90 ± 112.00	144.68	93.41–1005.02	0.787
GR [U/L]	96.07 ± 21.04	92.02	75.75–124.47	85.85 ± 40.45	83.58	1.60–185.29	0.308

**Table 7:** Oxidative stress parameters in regard to the umbilical cord blood of newborns delivered by caesarean section (CS) and the clinical condition of the newborn (Apgar score) at the 1st, 3rd, and 5th minute of life.

	Apgar Score in 1st Minute of Life of Newborns Delivered by CS (*n*= 108)						
	<10 pts (*n* = 12)			10 pts (*n* = 96)			*p*-Value
Parameter	$\bar{x}$ ± SD	Median	Min–Max	$\bar{x}$ ± SD	Median	Min–Max	
TAS [mmol/L]	1.86 ± 0.47	1.79	1.18–2.63	1.75 ±0.32	1.72	1.01–2.83	0.555
SOD [U/mL]	190.76 ± 17.33	180.47	167.00–217.60	197.99 ± 17.77	202.46	162.78–281.41	0.441
Mn [µmol/L]	0.08 ±0.04	0.07	0.04–0.16	0.15 ± 0.22	0.08	0.01–1.81	0.327
Cu [µmol/L]	7.57 ± 2.01	8.25	4.30–10.40	6.99 ± 4.90	6.10	0.09–33.40	0.177
Zn [µmol/L]	15.38 ± 9.81	14.50	2.40–32.20	13.38 ± 8.48	11.80	1.20–50.40	0.617
GPx [U/g Hg]	131.18 ± 18.76	129.93	96.92–161.53	152.01 ± 96.18	143.27	101.84–1057.14	0.089
GR [U/L]	90.14 ± 35.52	83.84	31.65–145.81	81.58 ± 39.39	72.20	1.24–191.69	0.352
	**Apgar Score in 3rd Minute of Life of Newborns Delivered by CS (*n*= 108)**						
	**<10 pts (*n* = 8)**			**10 pts (*n* = 100)**			***p*** **-Value**
**Parameter**	$\bar{x}$ **± SD**	**Median**	**Min–Max**	$\bar{x}$ **± SD**	**Median**	**Min–Max**	
TAS [mmol/L]	1.88 ±0.33	1.79	1.53–2.31	1.75 ± 0.34	1.72	1.01–2.83	0.424
SOD [U/mL]	190.52 ± 19.54	180.47	167.00–217.60	197.71 ± 17.69	201.35	162.78–281.41	0.562
Mn [µmol/L]	0.07 ± 0.02	0.06	0.04–0.10	0.14 ± 0.22	0.08	0.01–1.81	0.250
Cu [µmol/L]	7.55 ± 2.05	7.80	4.30–10.40	7.02 ± 4.82	6.15	0.09–33.40	0.337
Zn [µmol/L]	15.08 ± 12.56	7.60	2.40–32.20	13.48 ± 8.37	11.80	1.20–50.40	0.631
GPx [U/g Hg]	124.73 ± 19.82	129.23	96.92–155.92	151.49 ± 94.25	142.57	101.84–1057.14	0.063
GR [U/L]	89.83 ± 32.68	94.33	31.65–133.19	81.95 ± 39.16	72.20	1.24–191.69	0.447
	**Apgar Score in 5th Minute of Life of Newborns Delivered by CS (*n*= 108)**						
	**<10 pts (*n* = 5)**			**10 pts (*n* = 103)**			***p*** **-Value**
**Parameter**	$\bar{x}$ **± SD**	**Median**	**Min–Max**	$\bar{x}$ **± SD**	**Median**	**Min–Max**	
TAS [mmol/L]	1.95 ±0.45	2.22	1.32–2.31	1.76 ±0.33	1.72	1.01–2.83	0.226
SOD [U/mL]	179.86 ± 10.26	180.47	167.00–192.10	197.89 ±17.76	202.46	162.78–281.41	0.020
Mn [µmol/L]	0.07 ± 0.03	0.07	0.04–0.10	0.14 ± 0.22	0.08	0.01–1.81	0.285
Cu [µmol/L]	7.78 ± 1.43	7.80	6.00–9.50	7.02 ± 4.79	6.15	0.9–33.40	0.161
Zn [µmol/L]	15.07 ± 9.38	9.30	7.60–28.30	13.51 ± 8.59	11.80	1.20–50.40	0.912
GPx [U/g Hg]	134.38 ±20.60	129.93	111.67–161.53	150.65 ±93.47	141.17	96.92–1057.14	0.358
GR [U/L]	71.48 ±26.18	76.91	31.65–100.47	82.86 ±39.22	73.26	1.24–191.69	0.589

**Table 8:** PCA of the characteristics of the oxidative stress parameters in regard to umbilical cord blood for newborns assessed using the Apgar scale at the 1st minute of life.

Variables	Comp. 1	Comp. 2
Cu ^2^	0.25601444	0.40827393
Zn ^2^	−0.3735084	0.55592712
Mn	−0.1654171	−0.18012357
TAS ^1^	0.5457905	0.12727006
SOD ^1^	−0.4686455	−0.40252627
GR	−0.2705544	0.03880231
GPx	−0.1999567	−0.13219474
APGAR ^2^	0.3698925	−0.54277694

^1^ The first principal component correlates most strongly with TAS and SOD; ^2^ the second principal component correlates with Cu, Zn, and the Apgar score.

## Data Availability

The datasets analysed in the study are available via the RepOD platform: https://doi.org/10.18150/E9EUDQ.

## Abbreviations

The following abbreviations are used in this manuscript:

Tas	Total antioxidant status
GPx	Glutathione peroxidase
GR	Glutathione reductase
SOD	Superoxide dismutase
Zn	Zinc
Cu	Copper
Mn	Manganese
